# Supplementary material for: Excessive aggregation of fine particles may play a crucial role in adolescent spontaneous pneumothorax pathogenesis
Source: PeerJ. 2023 Nov 29;11:e16484. doi: 10.7717/peerj.16484 (PMC10693242; doi:10.7717/peerj.16484)
Supplement: Data S1 [file peerj-11-16484-s001.zip › Figure1.docx]

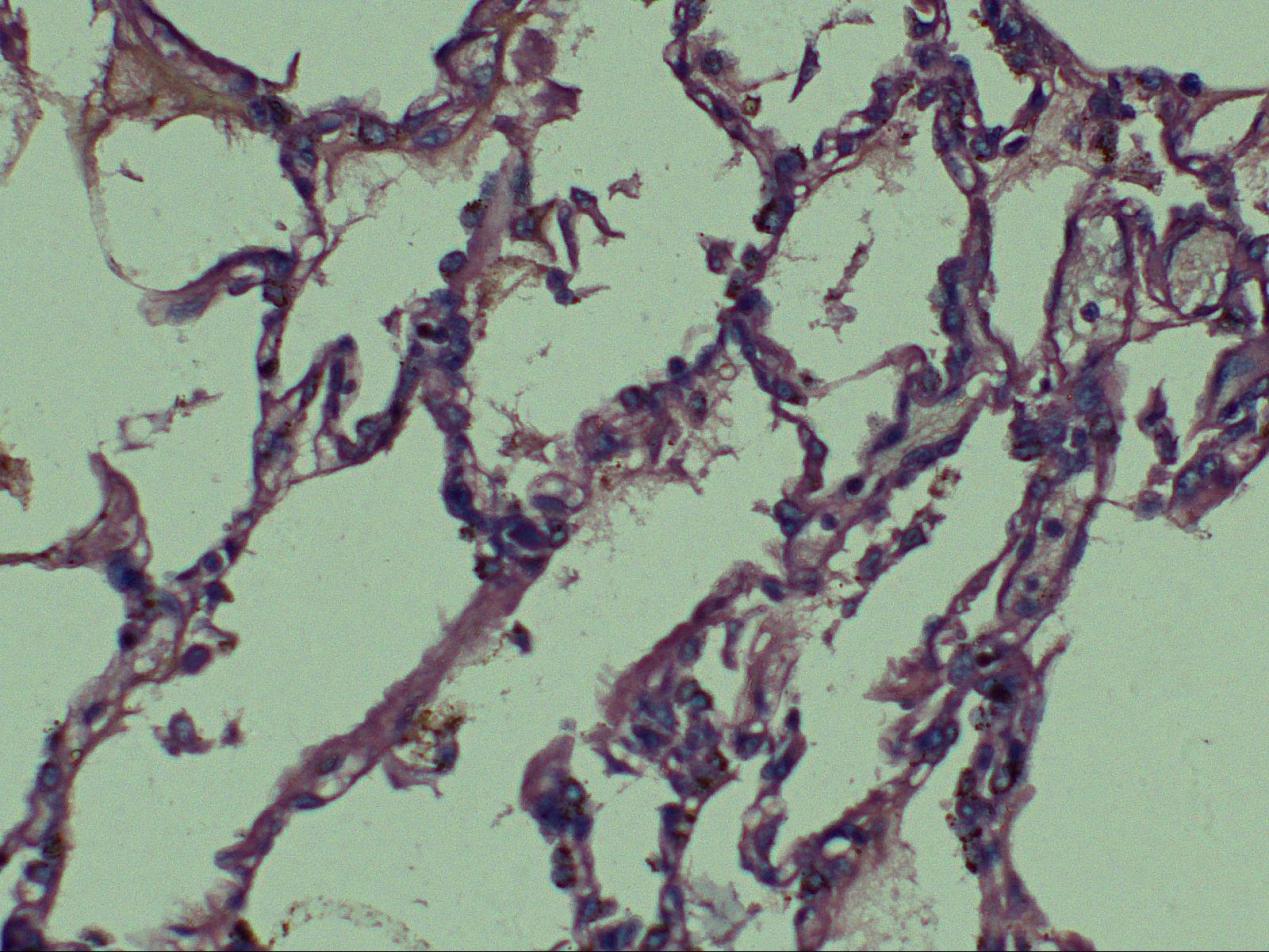


A 200×(S group,H&E)


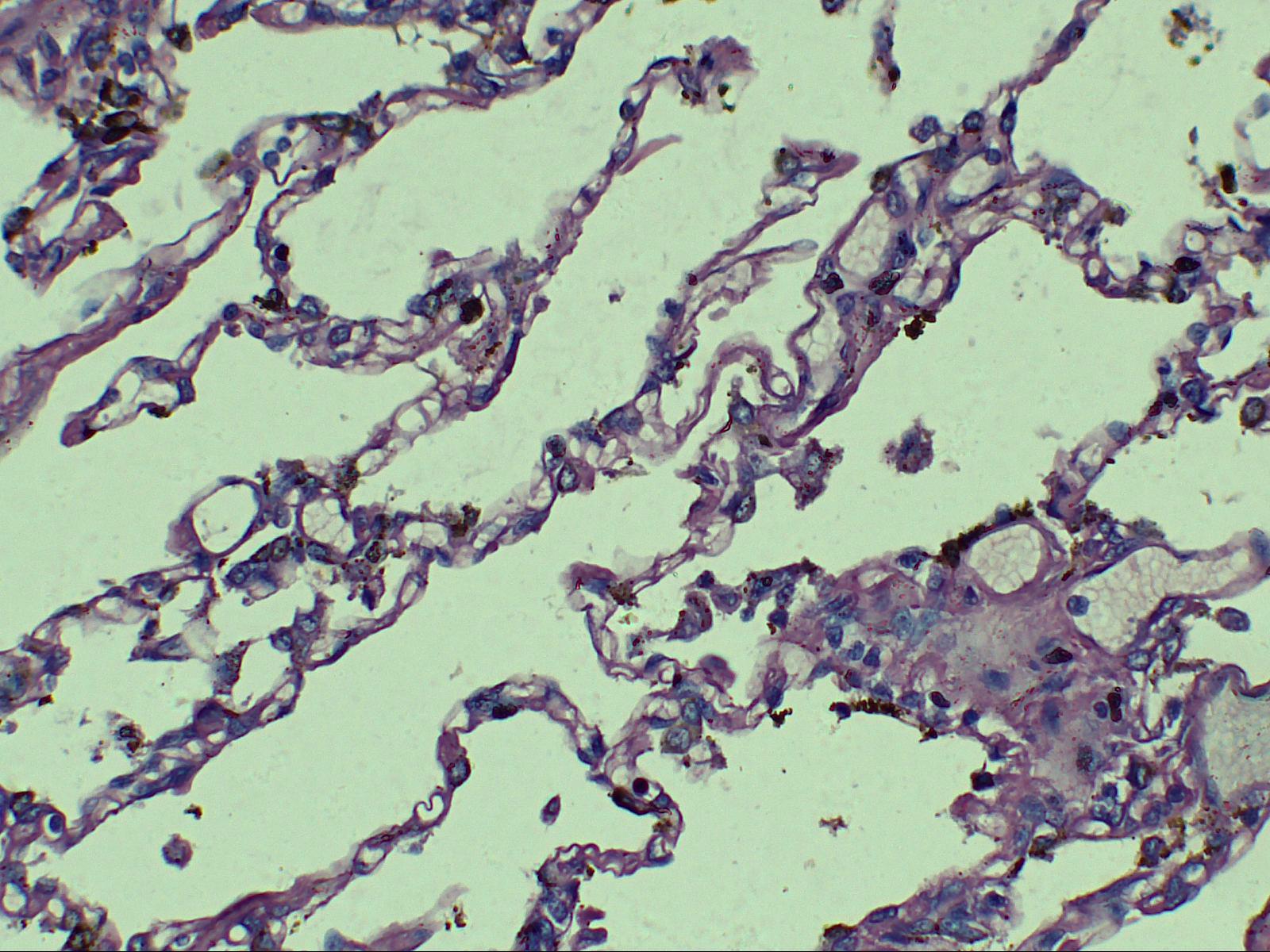


B 200×(N group,H&E)


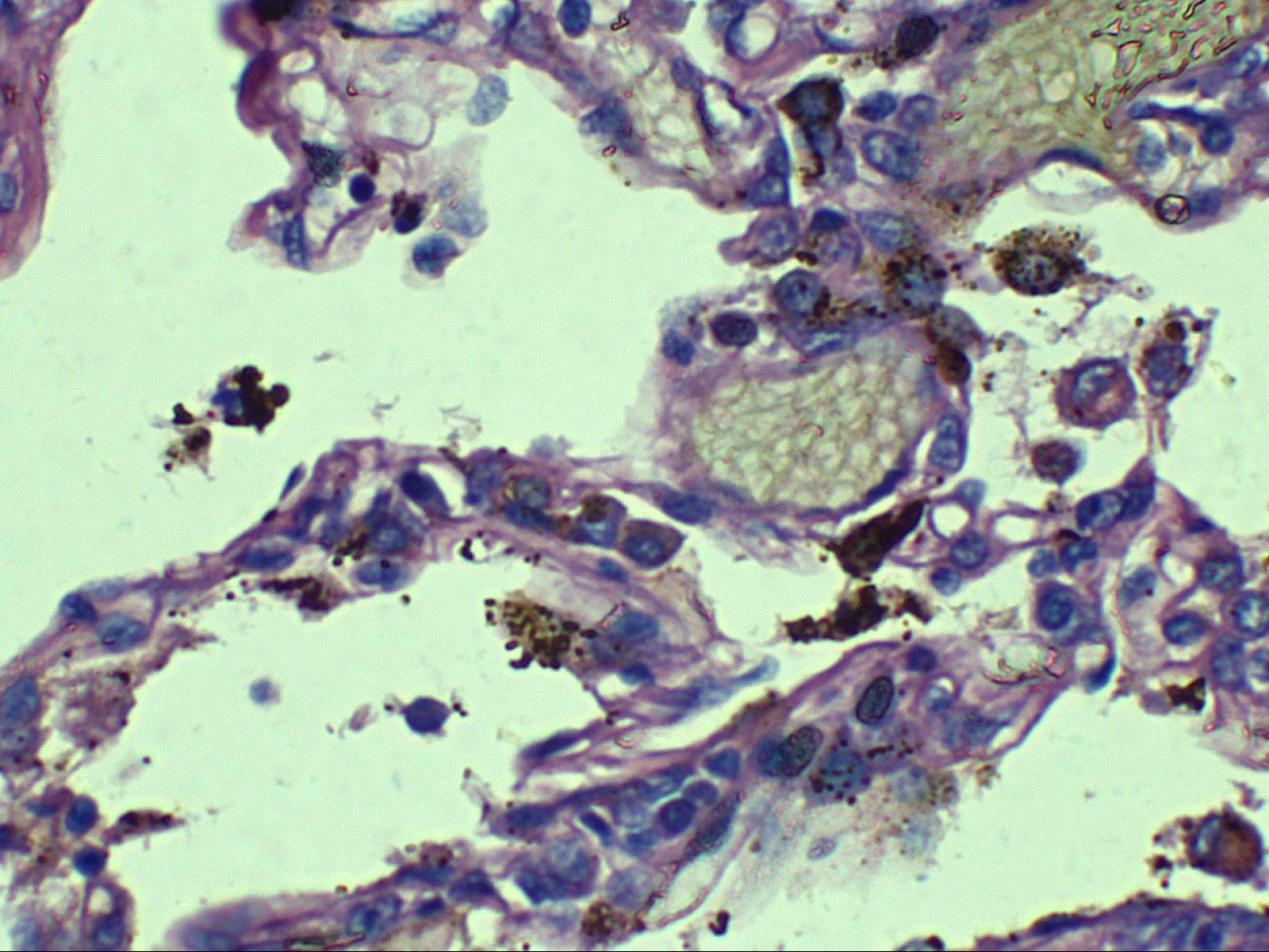


C 400×(B group,H&E)


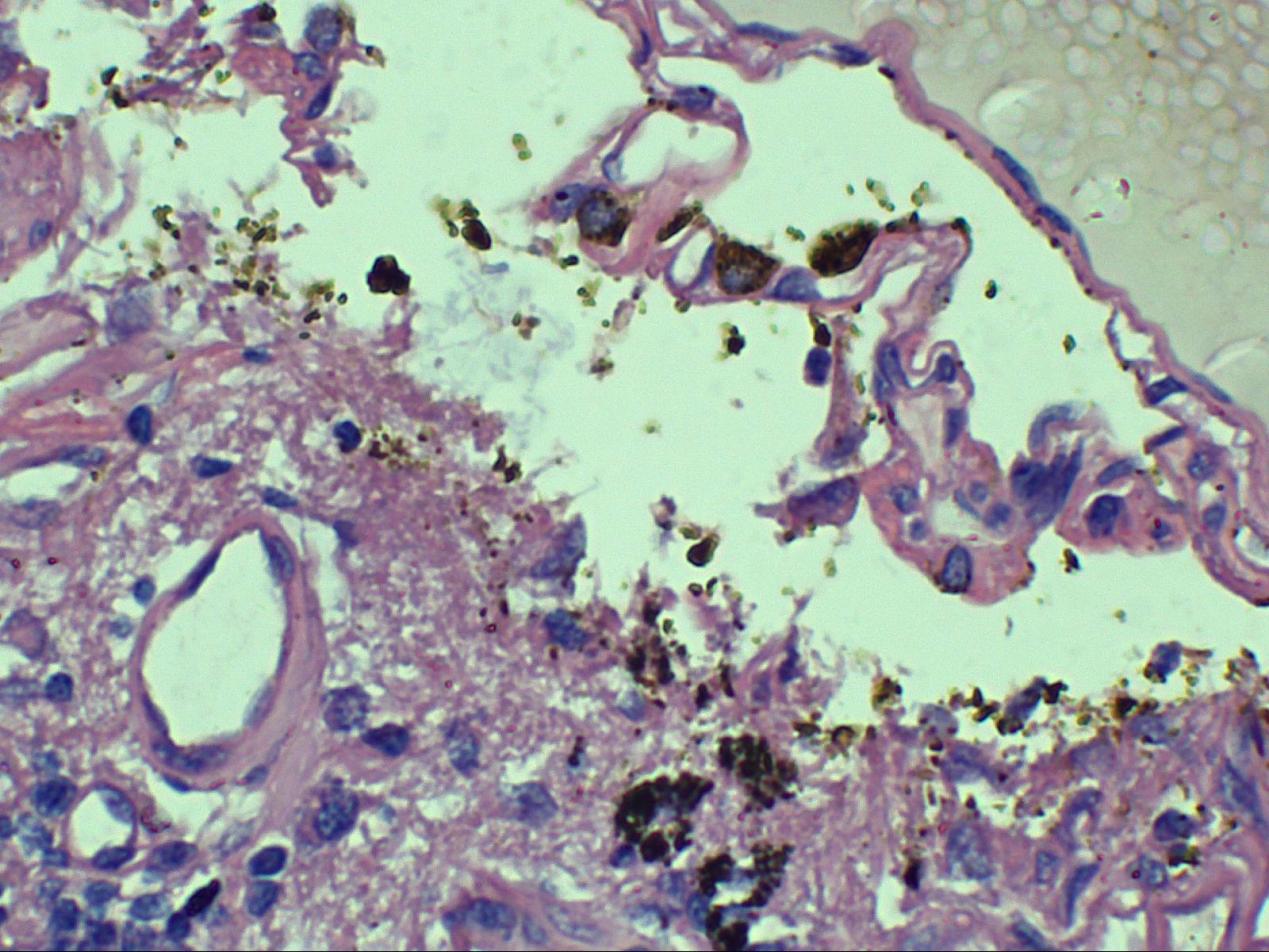


D 400×(B group,H&E)


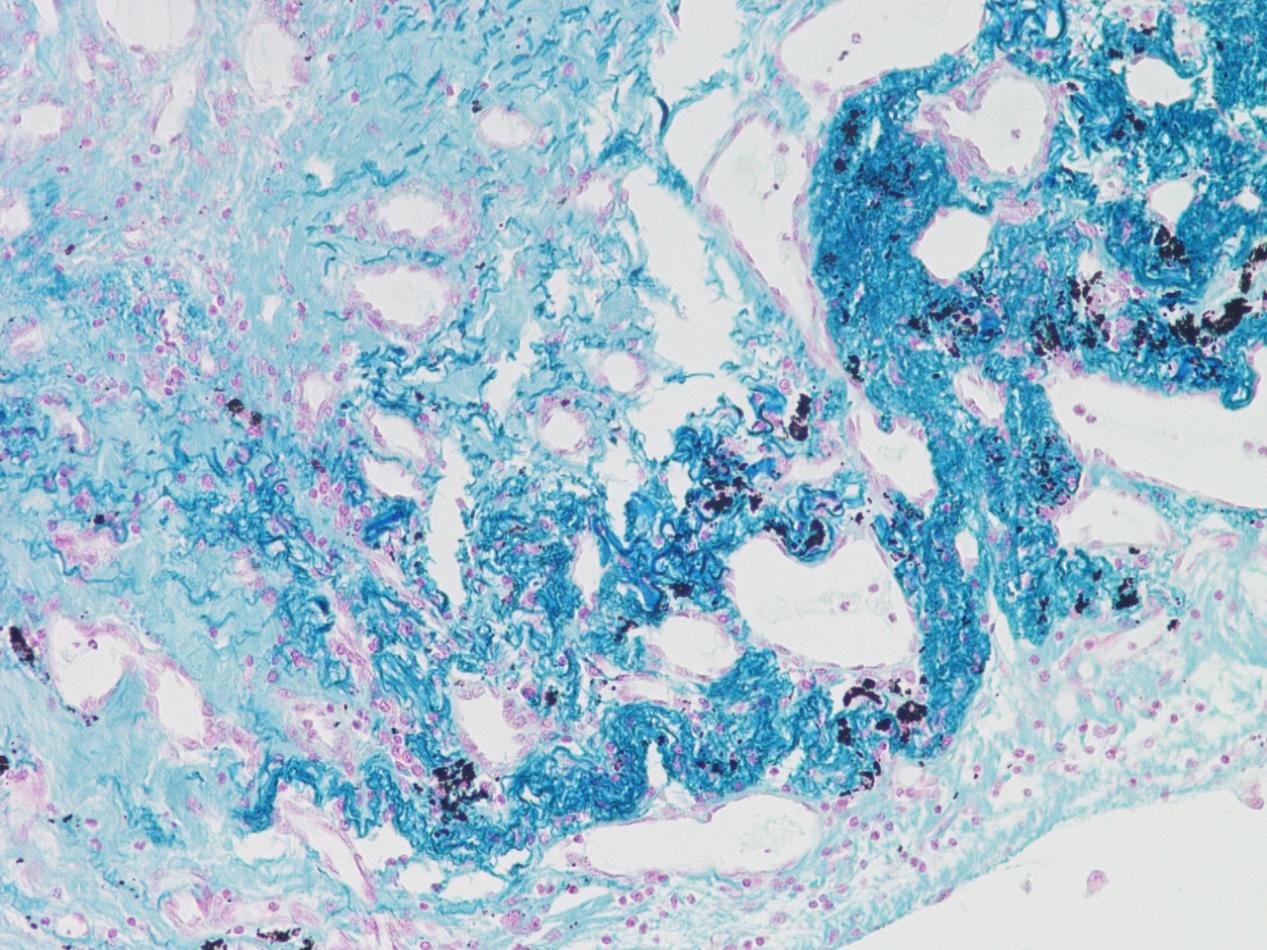


E 200×(B group,Victoria blue)


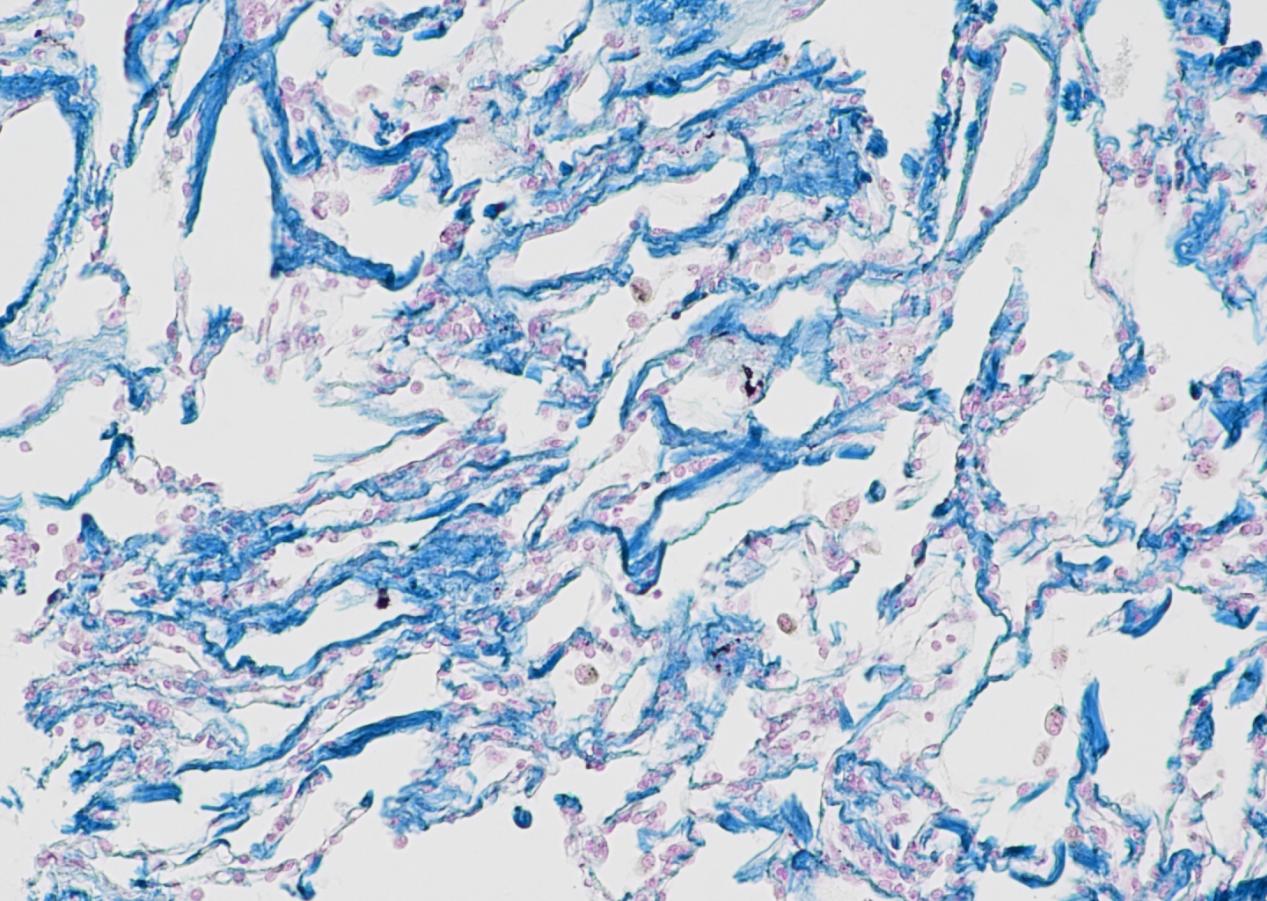


F 200×(B group,Victoria blue)


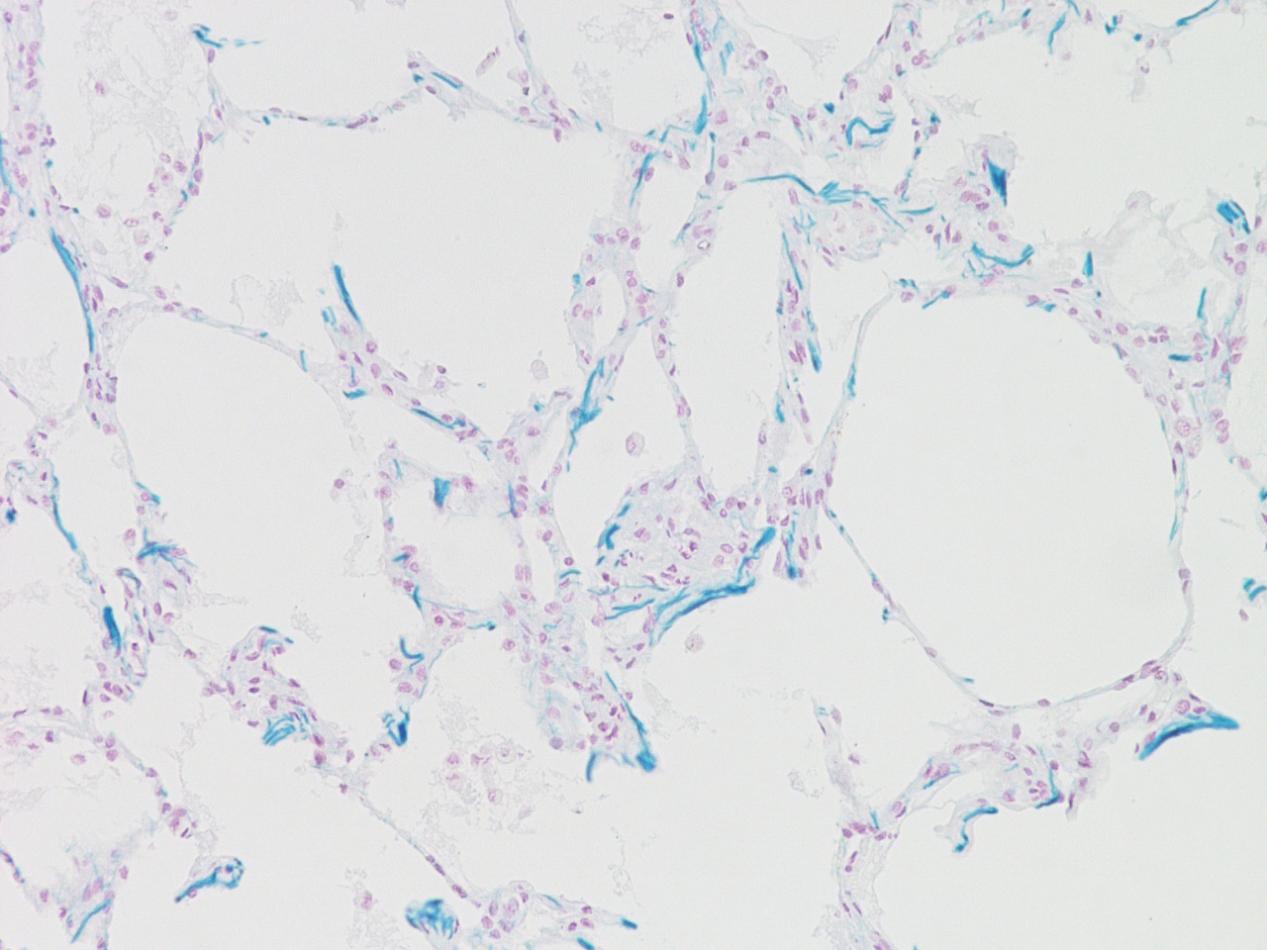


G 200×(S group,Victoria blue)


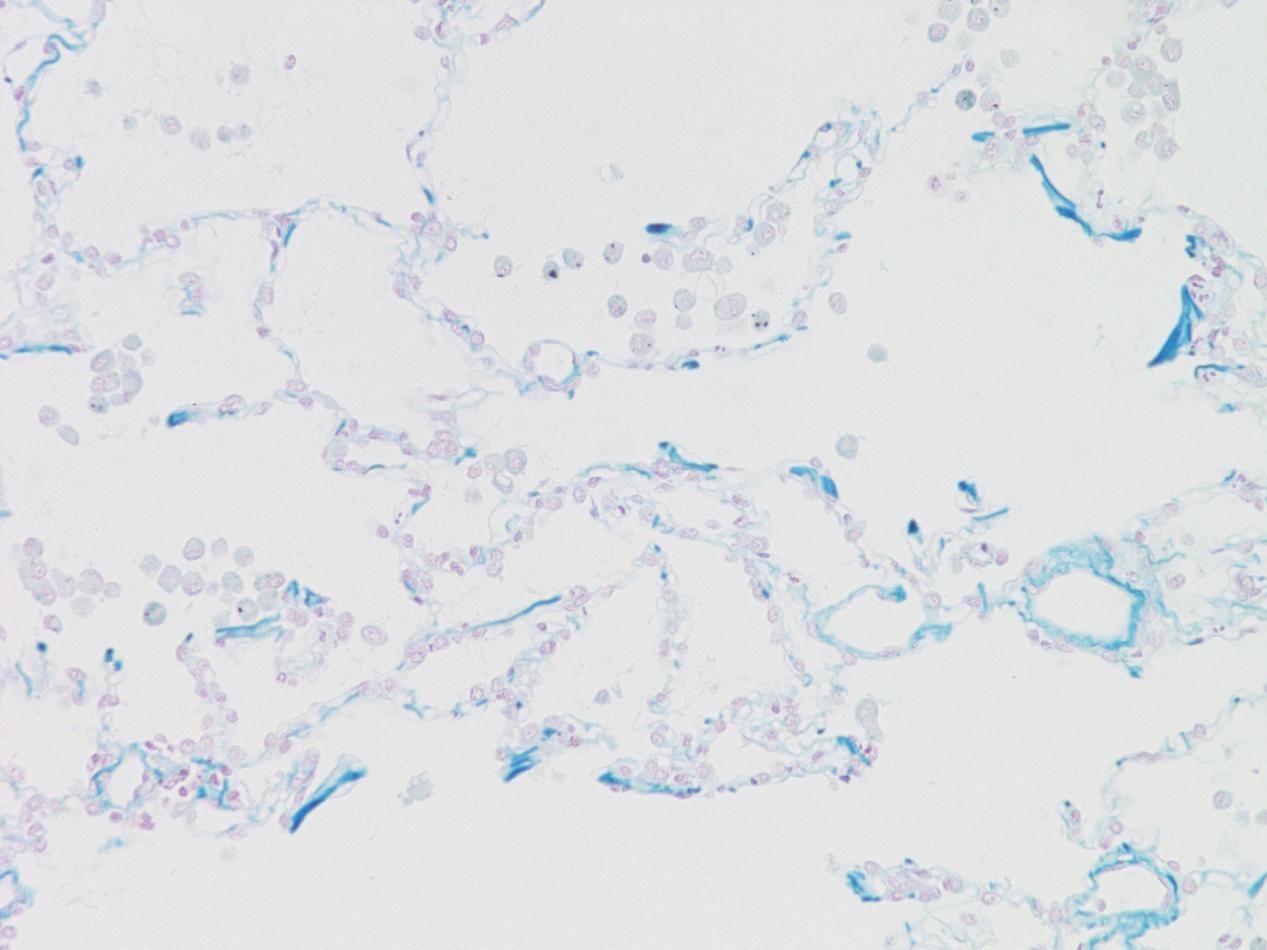


H 200×(N group,Victoria blue)
