# Supplementary material for: Excessive aggregation of fine particles may play a crucial role in adolescent spontaneous pneumothorax pathogenesis
Source: PeerJ. 2023 Nov 29;11:e16484. doi: 10.7717/peerj.16484 (PMC10693242; doi:10.7717/peerj.16484)
Supplement: Data S1 [file peerj-11-16484-s001.zip › Figure2.docx]

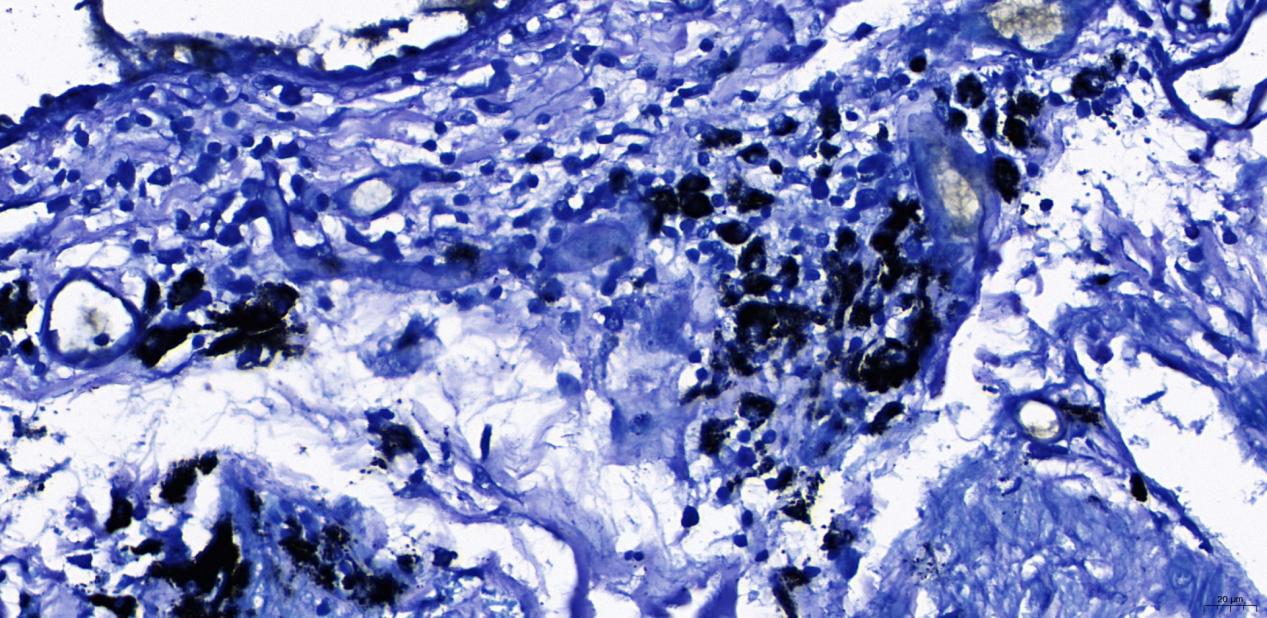


A 400(B group,W-G)


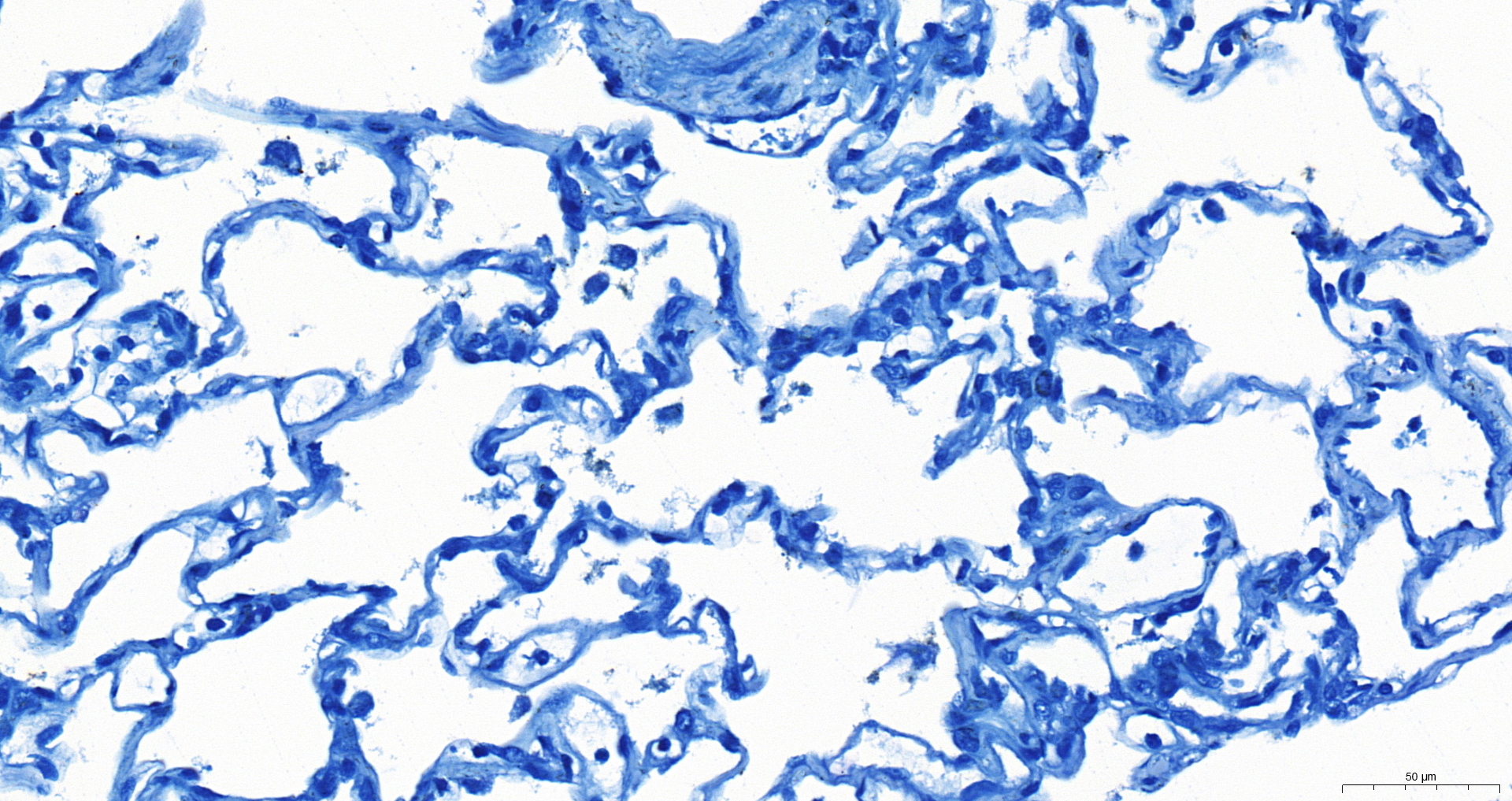


B 400(S group,W-G)


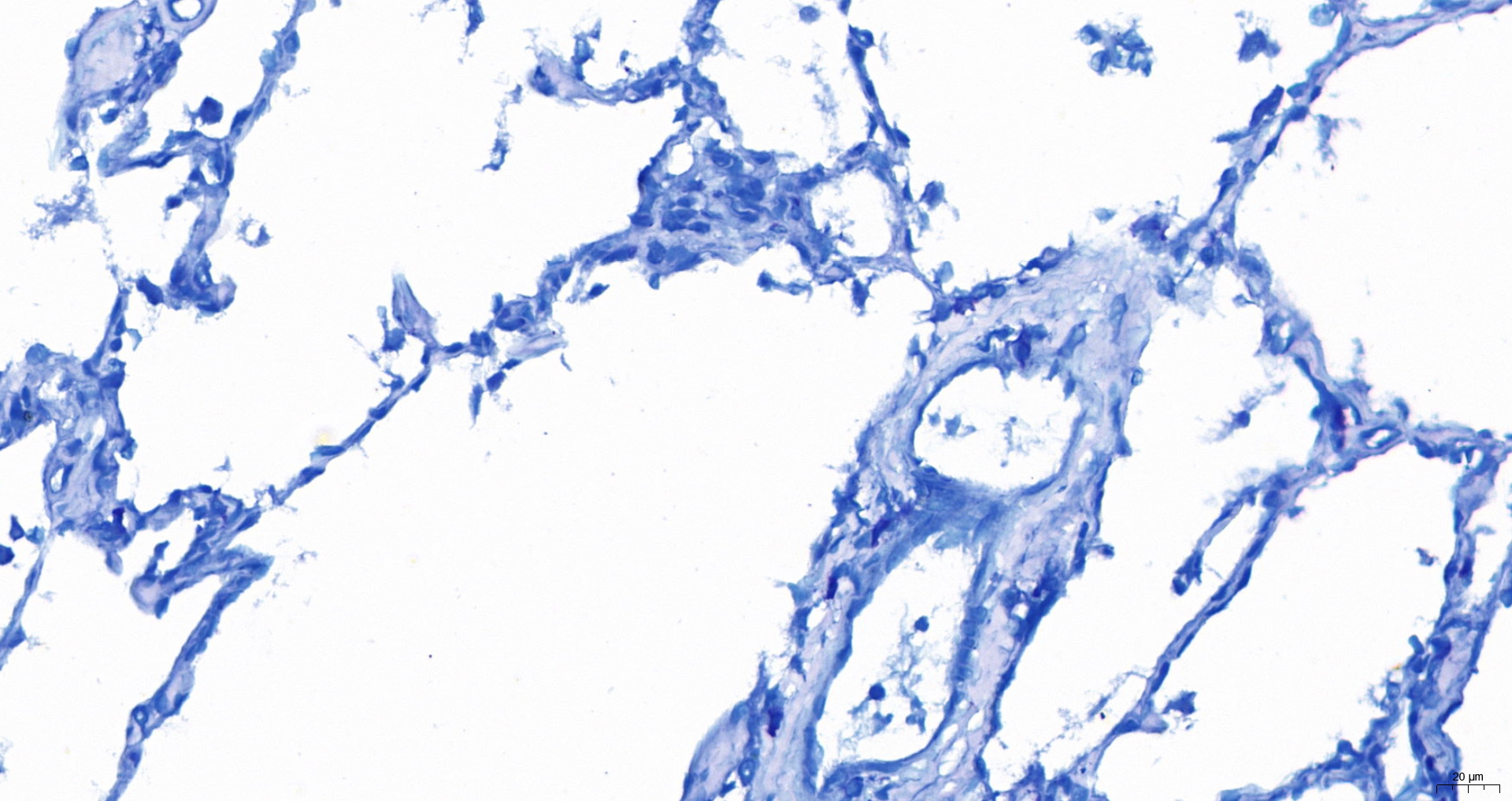


C 400(N group,W-G)


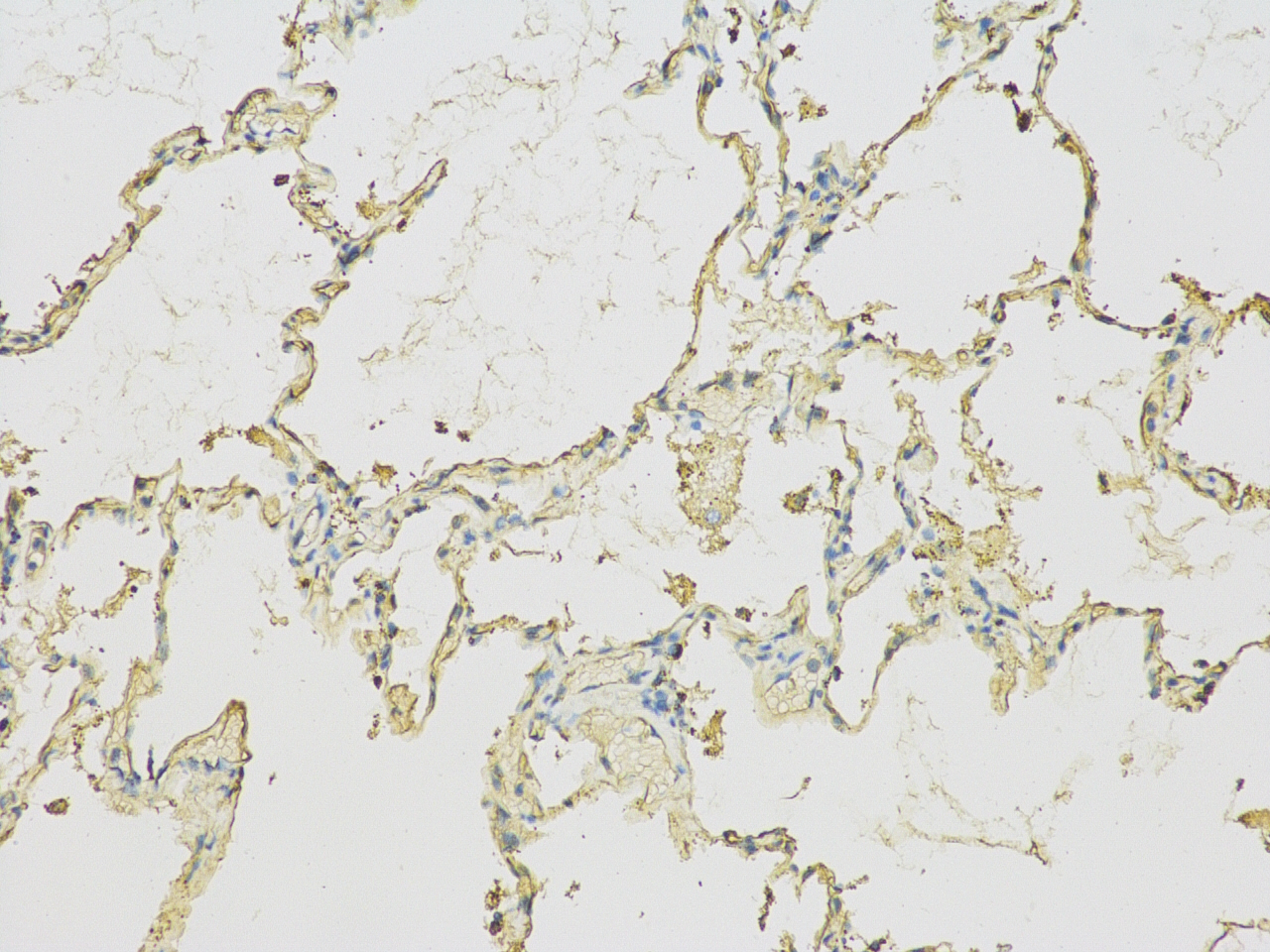


D 200(B group,MCP-1)


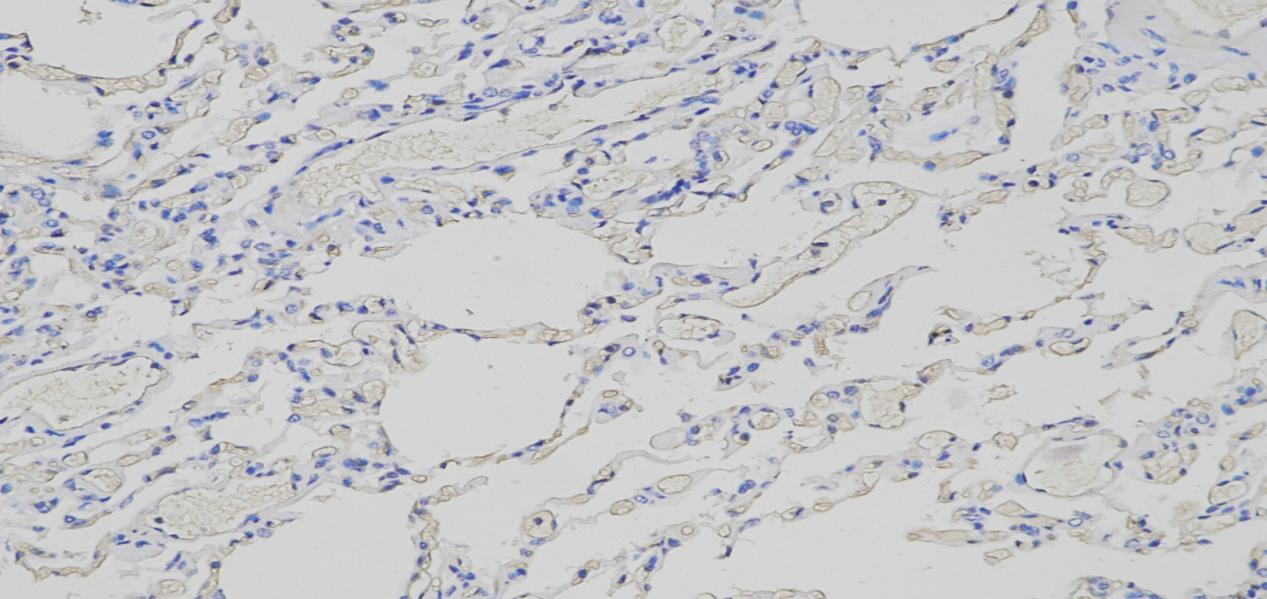


E 200(S group,MCP-1)


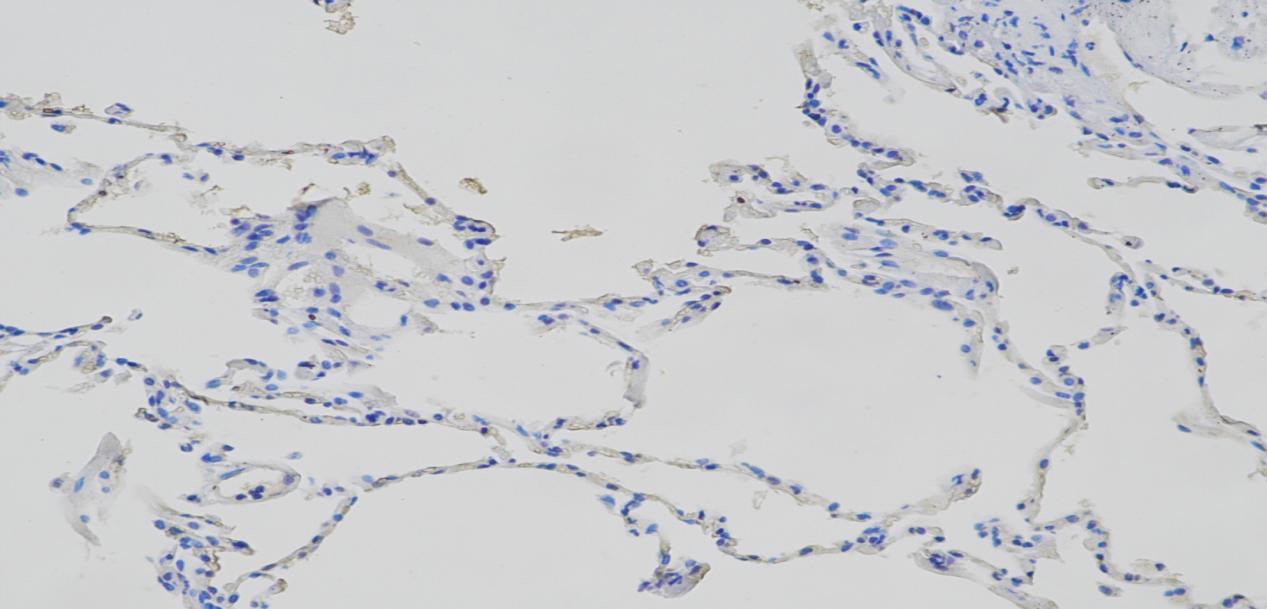


F 200(N group,MCP-1)


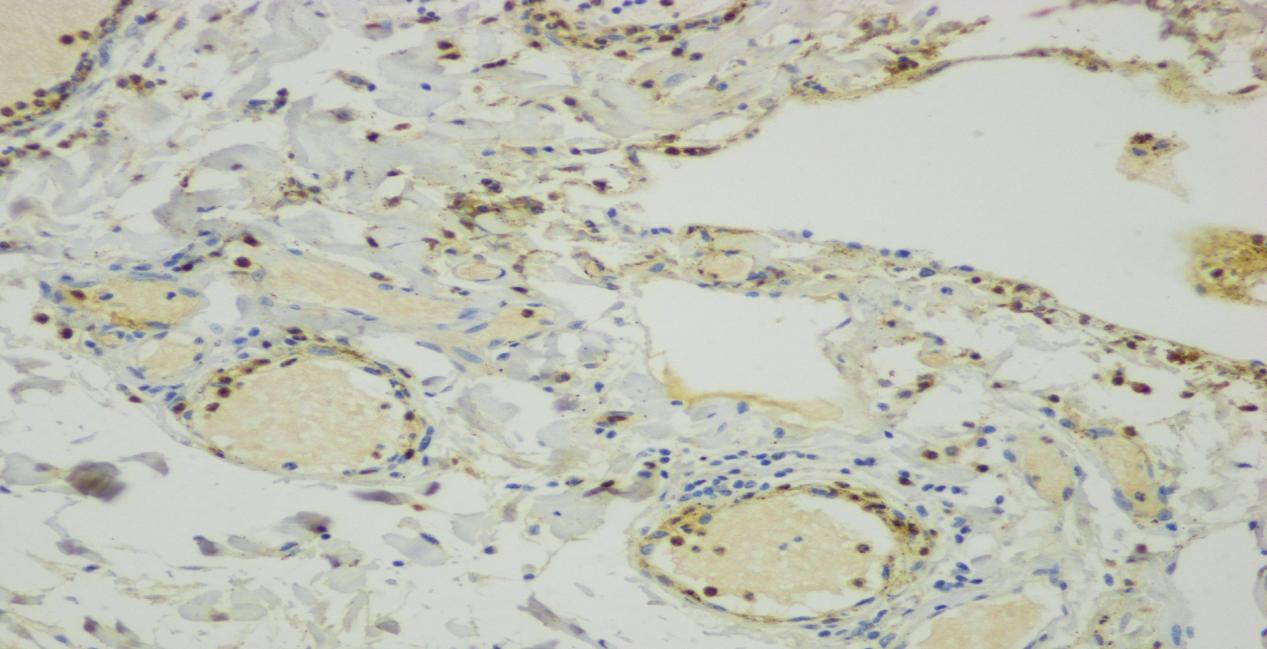


G 200(B group,MMP-9)


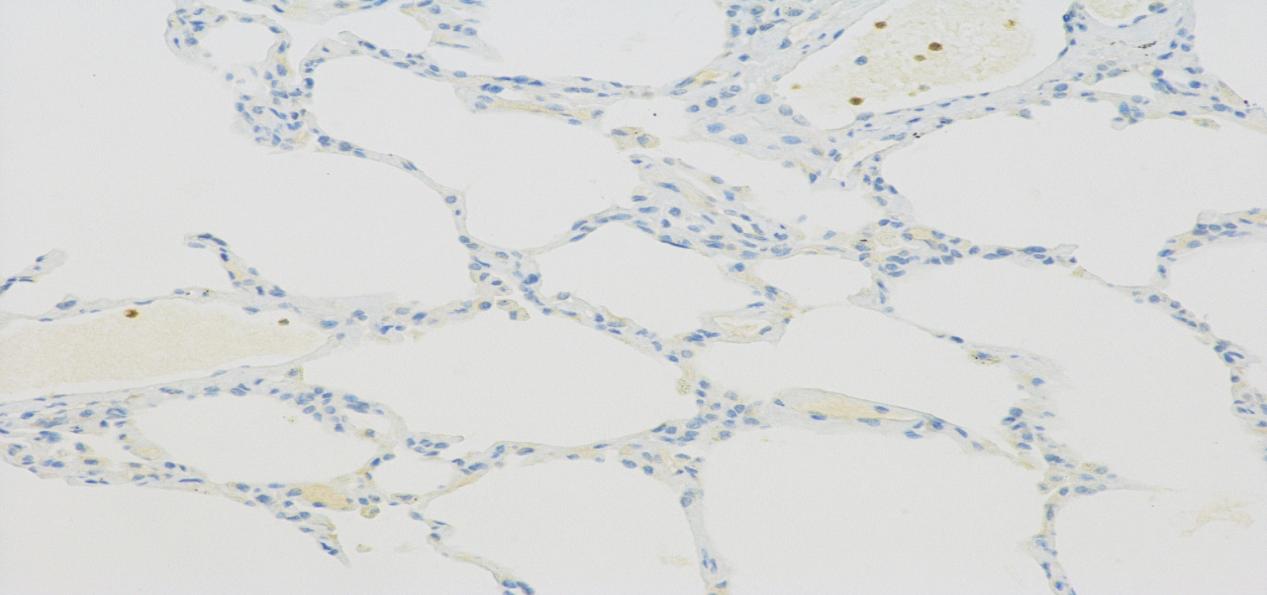


H 200(S group,MMP-9)


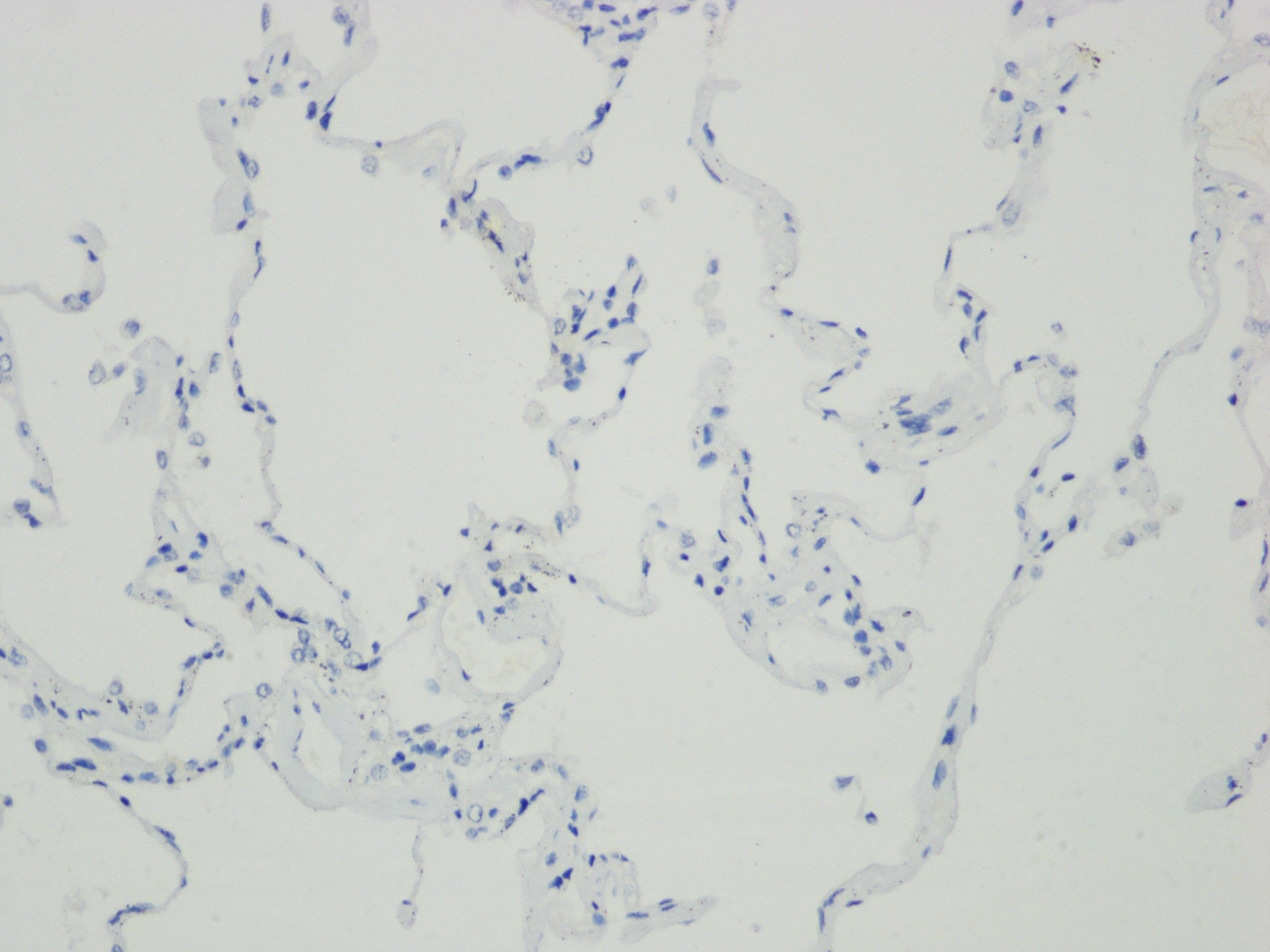


I 200(N group,MMP-9)
