# Supplementary figures and images for: Excessive aggregation of fine particles may play a crucial role in adolescent spontaneous pneumothorax pathogenesis
Source: PeerJ. 2023 Nov 29;11:e16484. doi: 10.7717/peerj.16484 (PMC10693242; doi:10.7717/peerj.16484)

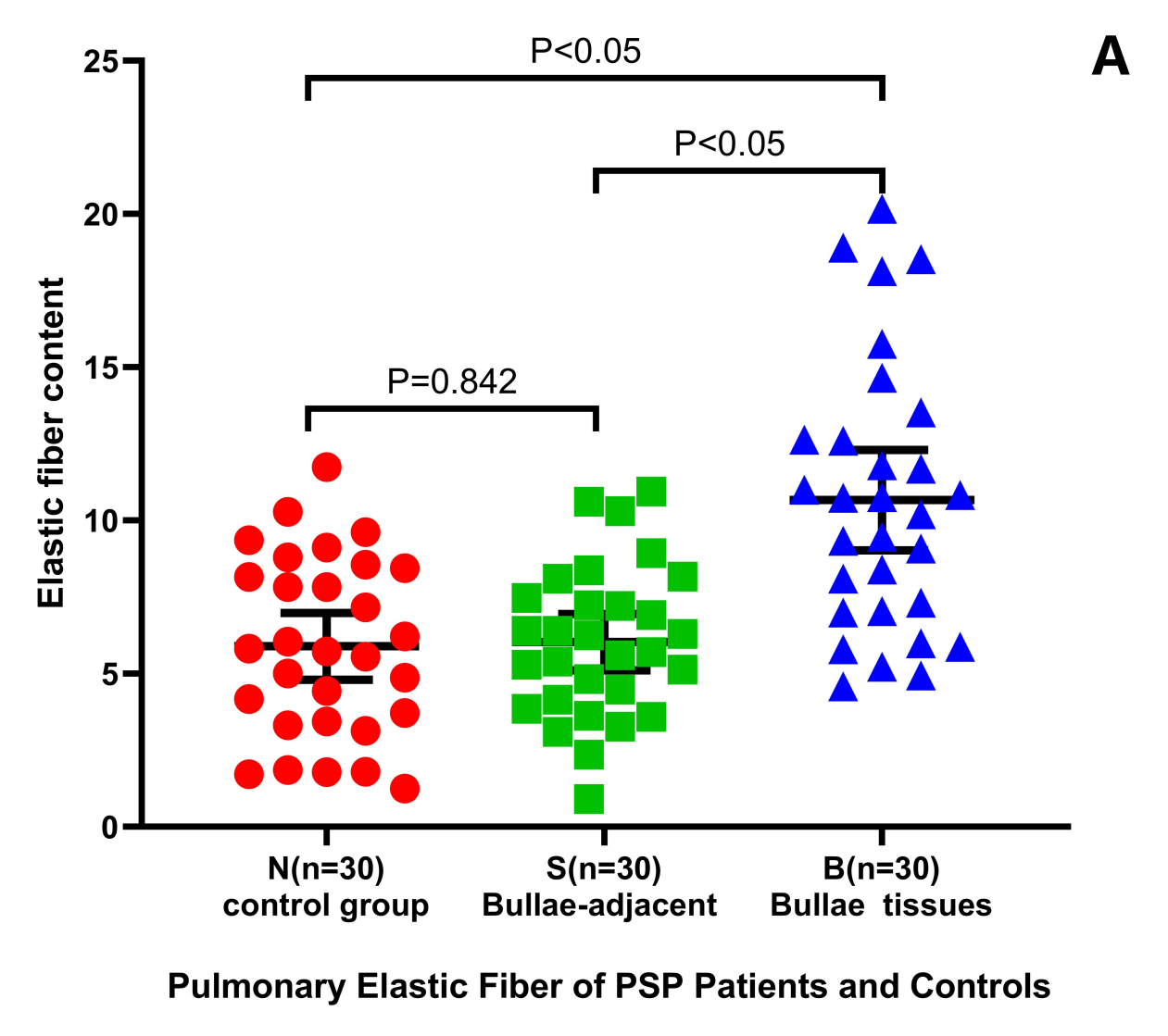

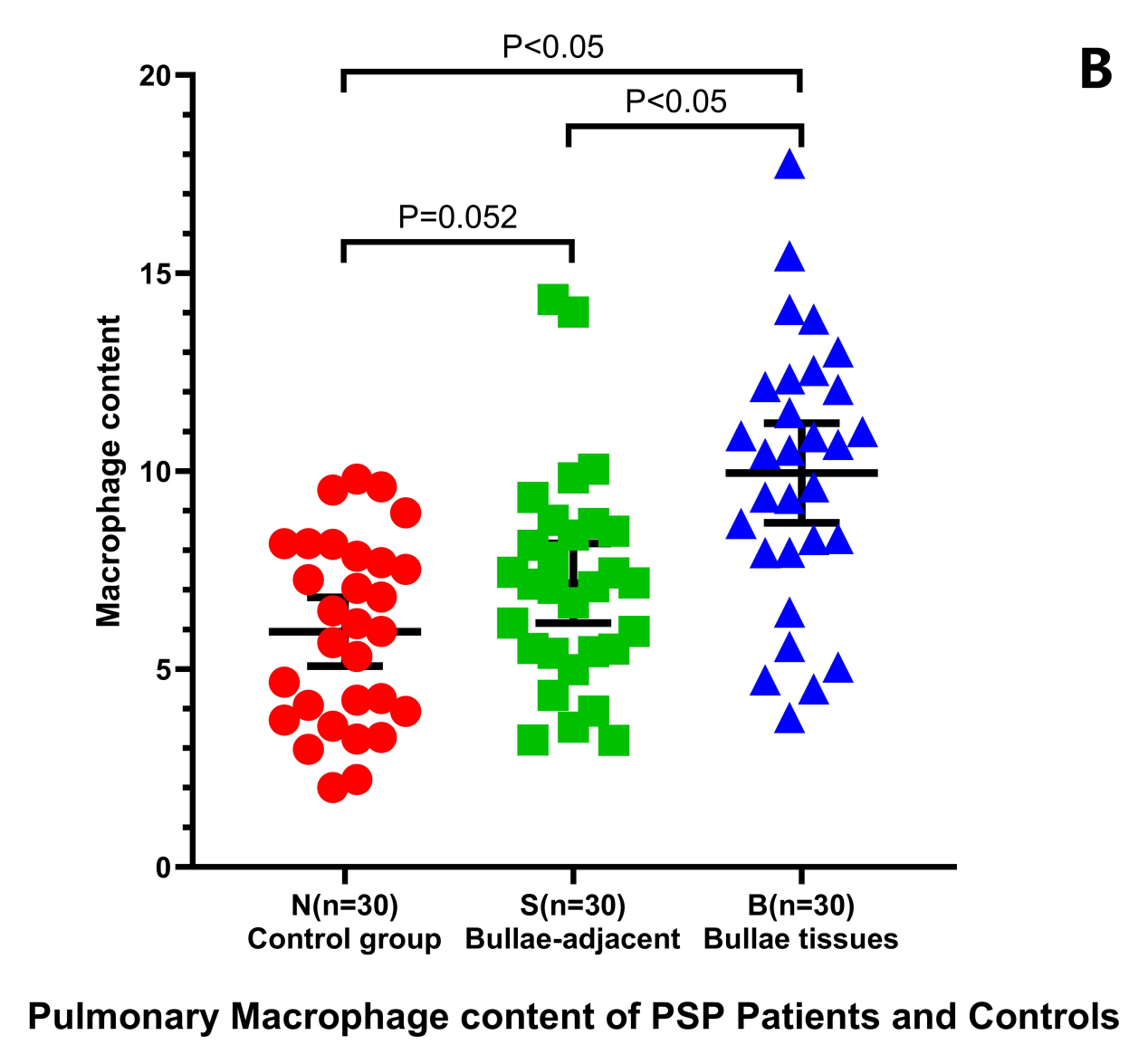

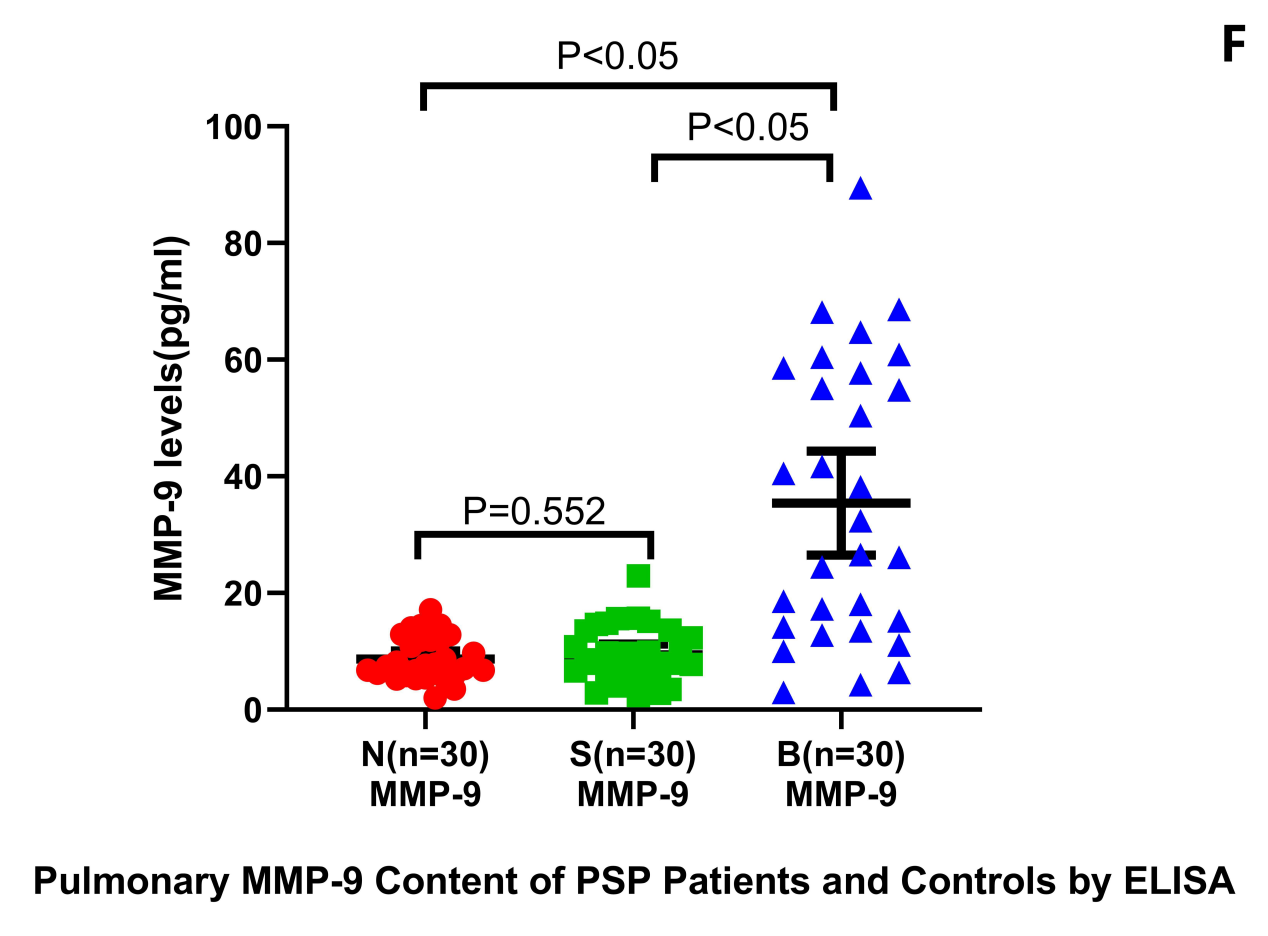

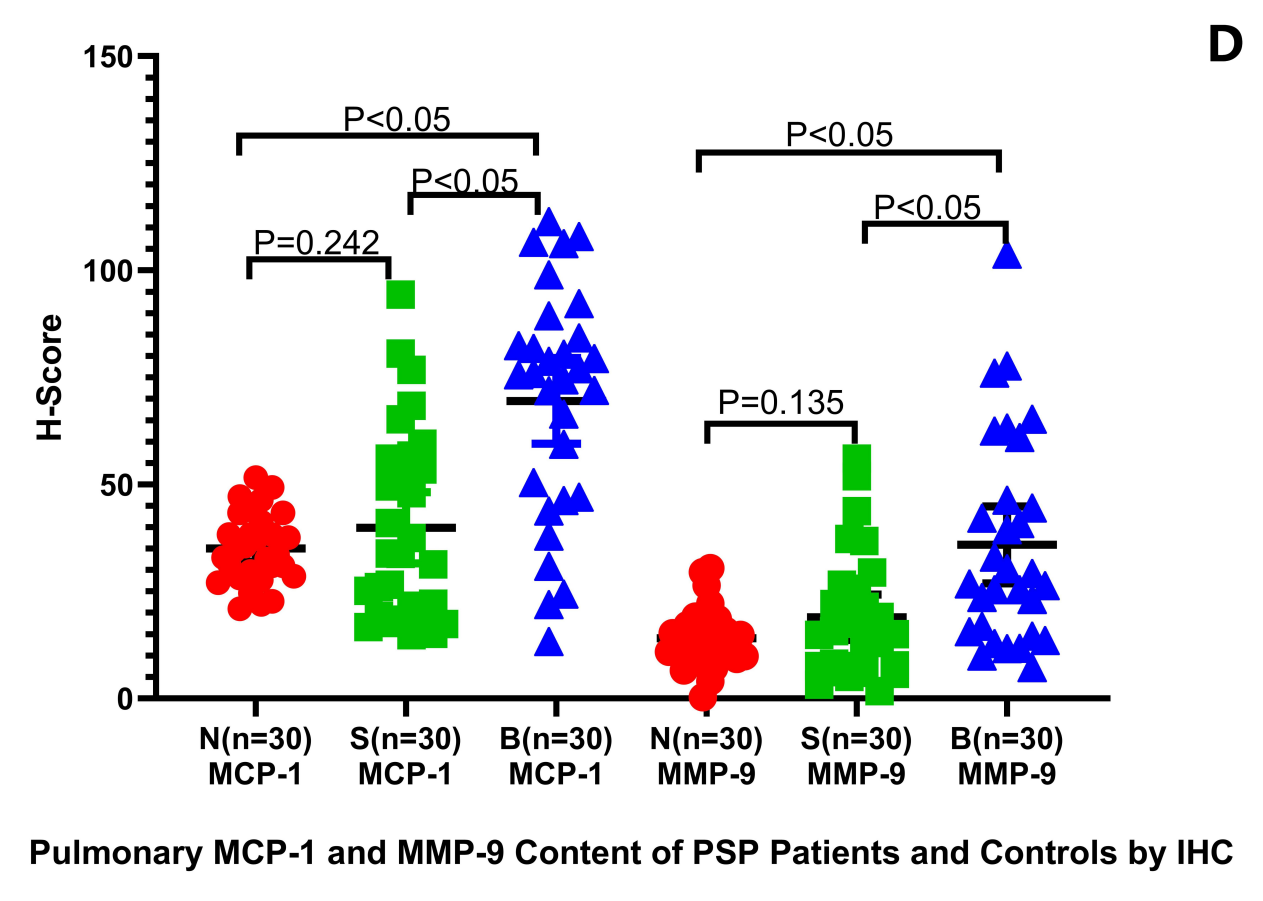

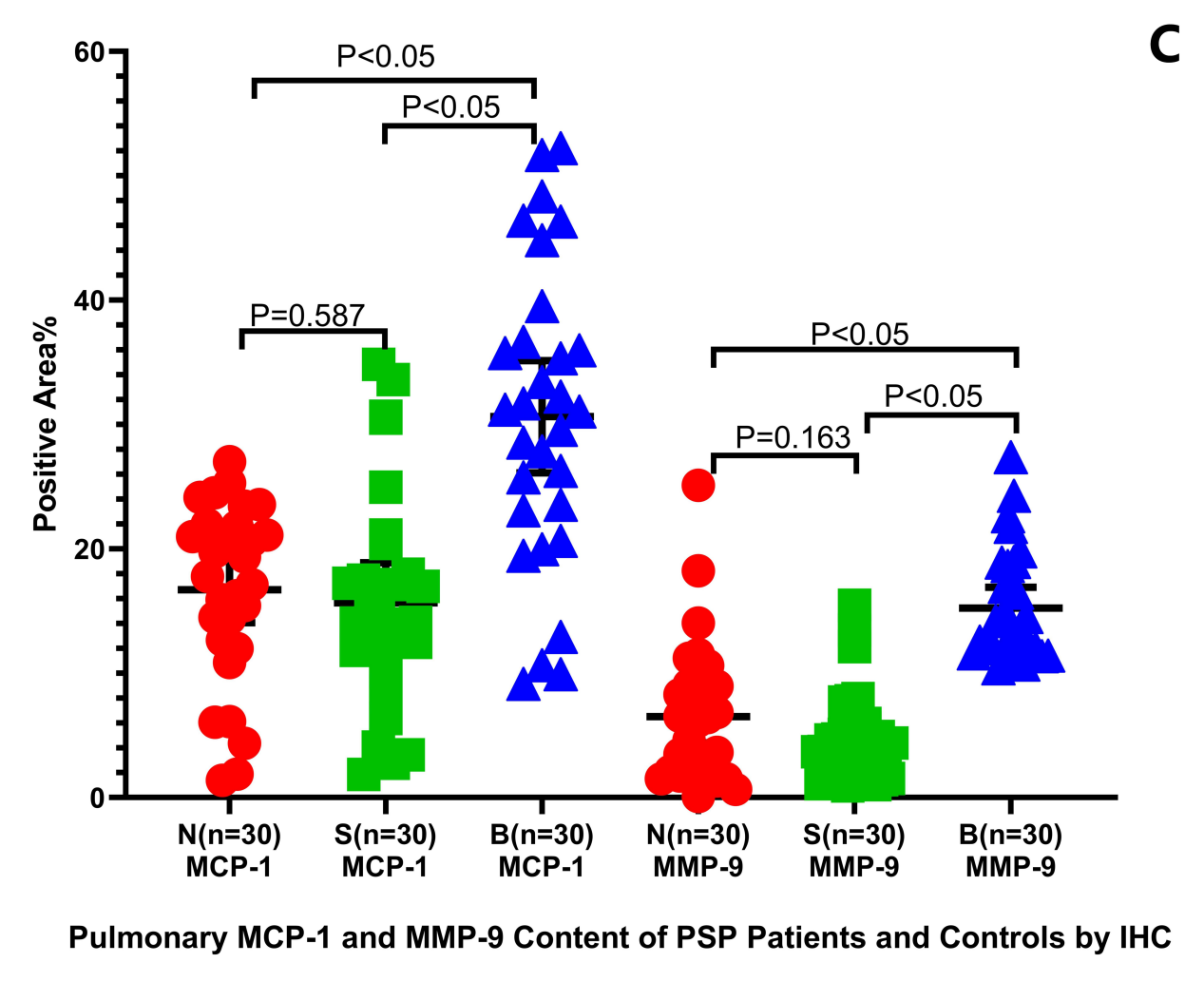

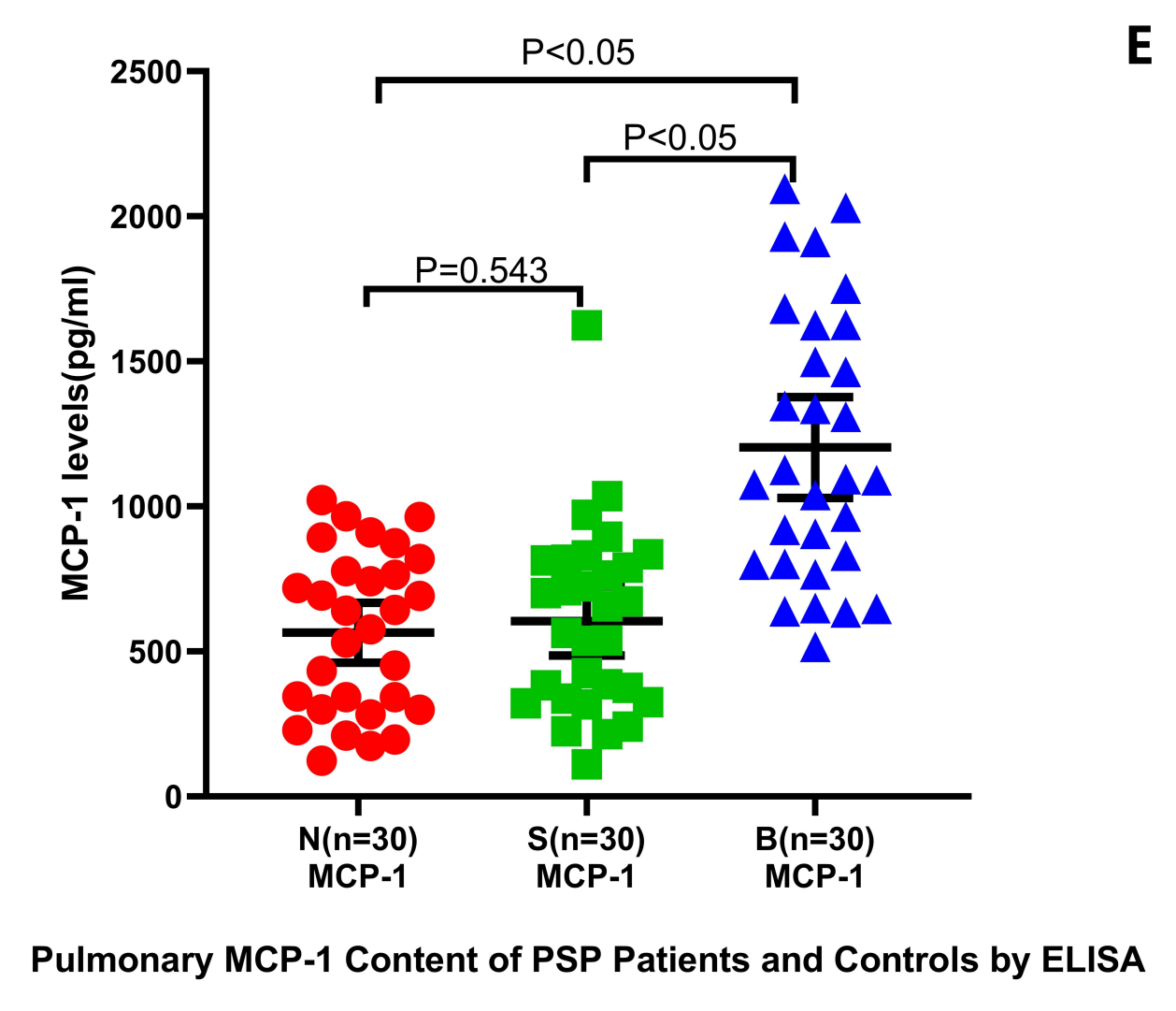

Supplement: Data S1 [file peerj-11-16484-s001.zip › Figure3 and 4.docx]
